# An Act for the Regulation of the Practice of Dentistry in the State of Michigan

**Published:** 1894-08

**Authors:** 


### An Act for the Regulation of the Practice of Dentistry in the State of Michigan.

SECTION I. It shall hereafter be unlawful for any person to practice dentistry in this State unless he shall first have obtained a certificate of enregistration from the Board of Dental Examiners provided for by this Act, and filed the same or a certified copy thereof, with the County Clerk of the County in which he practices, all as hereinafter provided. The provisions of this act shall in no way apply to or affect any person who is now located and lawfully in actual practice in this State.

SEC. II. Said Board of Dental Examiners shall be appointed by the Governor of the State, and shall consist of five practical dentists who shall be regular graduates of a reputable dental college, duly incorporated under the laws of this or some other State of the United States. The members of the first board under the provisions of this Act shall consist of the members of the present Board of Dental Examiners, who shall hold their offices as members of such new board for the term for which they were appointed under said former act, and until their successors are duly appointed. All vacancies in said board shall be filled by appointment by the governor as hereinafter provided.

The term for which members of said board shall be appointed shall be three years and until their successors shall be duly appointed. It is also hereby provided that no person shall serve to exceed two terms in succession.

SEC. III. The Board of Examiners at its first regular meeting, annually, shall choose a President, a Secretary and a Treasurer from its members who severally shall have the power during their term of office to administer oaths and take affidavits, certifying thereto under their hands and seal of the said board, and to receive, disburse and take care of all moneys which come into the hands of the board.

SEC. IV. The Board of Examiners shall meet semi-annually for the purpose of examining applicants, after having given notice in the papers throughout the State for four weeks prior to each examination. The said board is authorized to incur all necessary expenses in the prompt and efficient discharge of its duties, and pay the same with any money in the hands of its treasurer. A majority of said board shall all times constitute a quorum. It is furthermore provided that in the event of any member of said board absenting himself from two of its regular meetings consecutively, the board may declare a vacancy to exist, which vacancy shall be filled by the means hereinbefore provided.

SEC. V. It shall be the duty of the first board, hereinbefore provided for, to meet and elect officers at its first meeting, and within ten days thereafter to transfer to a register to be provided by them for that purpose, the name, residence and place of business of each and every person who, pursuant to an Act of the Legislature of the State of Michigan, (approved May 26th, 1891), shall be duly registered on the books of the board created by said act of May 26th, 1891. No certificates of license to practice dentistry shall be issued after the date the board transfers names, etc. It shall be the duty of the said secretary of the first board hereby created to send to each person so registered, prior to the date the law takes effect, a certificate of his enregistration, signed and certified thereto by the president and secretary, under their hands and seal of said board.

SEC. VI. Any person or persons who shall desire to begin the practice of dentistry in the State of Michigan on and after the date the law takes effect, shall file their names together with an application for examination with the Secretary of the State Board of Dental Examiners, and at the time of making such applications shall pay to the treasurer of said board a fee of ten dollars ($10.00) and shall present themselves at the first regular meeting thereafter of said board to undergo examination before that body. The examination shall be elementary and practical in character but sufficiently thorough to test the fitness of the candidates to practice dentistry. It shall include, written in the English language, questions on the following subjects: Anatomy, Physiology, Chemistry, Materia Medica, Therapeutics, Metallurgy, Histology, Pathology, Operative and Surgical Dentistry, Mechanical Dentistry, Bacteriology, Oral Surgery and also demonstrations of their skill in Operative and Mechanical Dentistry. All persons successfully passing such examinations shall be registered as licensed dentists in the board register provided for in Sec. V., and also receive a certificate of such enregistration, said certificate to be signed and certified thereto by the president and secretary under their hands and seal of said board.

SEC. VII. Recipients of said certificate of enregistration shall present the same for record to the county clerk of the county in which they are to practice dentistry. Said clerk shall record said certificate in a book to be provided by him for that purpose and shall be paid a fee of twenty-five cents for registration of the same. A person so licensed, wishing to practice in another county, must obtain from the county clerk in which said certificate of registration is recorded a certified copy of such record, or else obtain a new certificate of enregistration from the Board of Examiners, and shall, before practicing in such county, file the same for record with the clerk of that county to which he removes and pay the clerk for recording the same a fee of twenty-five cents. Said board and clerk shall be entitled to a fee of fifty cents for the re-issue of any certificate, or making and certifying to a copy of the record of any such certificate. Any failure, neglect or refusal on the part of any person holding such certificate or copy of record to file the same for record as hereinbefore provided for six months from the issuance thereof shall forfeit the same.

SEC. VIII. All persons shall be said to be practicing dentistry within the meaning of this act who shall for fee or salary or other rewards paid either to themselves or to another person for operations or parts of operations of any kind, treat diseases or lesions of the human teeth or jaws, or correct mal-positions thereof; but nothing in this act contained shall be taken to apply to acts of bona fide students of dentistry done in pursuit of clinical advantages under the direct supervision of a preceptor or a licensed dentist in this State, or during the period of their enrollment in a dental college and furthermore, it shall be prima facie evidence of the practice of dentistry, if any person or persons advertise themselves as dentists or hold themselves out for the practice of dentistry in any manner whatsoever by sign or otherwise, provided their certificate is not recorded as hereinbefore provided.

SEC. IX. Any person who shall practice dentistry in this State in violation of the provision of this Act, shall be deemed guilty of a misdemeanor and upon conviction thereof shall be fined not less than five ($5.00) dollars, nor more than one hundred ($100.00) dollars, or sentenced to imprisonment in the county jail for a period not exceeding ninety (90) days or both ; such fine and imprisonment is in the discretion of the Court; provided that nothing in this Act shall be construed so as to interfere with physicians and surgeons in their practice as such.

SEC. X. Out of the funds coming into possession of the board the members of said board shall receive as compensation the sum of three ($3.00) dollars for each day actually engaged in the duties of their office, and mileage at the rate of three (3c.) cents per mile for all distance necessarily traveled in going to and coming from meetings of the board. Said expenses shall be paid from fees and assessments received by the board under the provision of this Act, and no part of the salary or other expenses of the board shall ever be paid out of the State treasury. Any sum in excess of one hundred ($100.00) dollars which shall under the provisions of this Act accumulate in the treasury of said board, shall be paid by the treasurer thereof into the State treasury.

SEC. XI. Any person who shall knowingly or falsely claim or pretend to have or hold a certificate of enregistration, diploma or degree granted by a society or by said board, or who shall falsely and with the intent to deceive the public, claim or pretend to be a graduate from any incorporated dental college, not being such graduate, shall be deemed guilty of a misdemeanor and shall be liable to the penalties provided in Sec. IX of this Act.

SEC. XII. Any person who shall be licensed under the provisions of this Act and who shall practice dentistry under a false name, with intent to deceive the public, shall be liable to have said license revoked upon twenty days' notice of such proposed revocations and of the time and place of considering such revocations, by order of the State Board of Dental Examiners. And any person who after revocation of their license shall continue to practice dentistry in the State of Michigan shall be deemed guilty of the violation of the provisions of this Act, and shall be subject to the penalties provided therein. Nor shall a certificate to a person under one name be any defense to an action brought against them for practicing without a certificate under another.

SEC. XIII. It shall be the duty of the respective County Prosecuting Attorneys to prosecute all violations of this Act. In case of the failure of the prosecutor to act, it shall be the duty of the Court to appoint an Attorney to conduct such prosecutions.

SEC. XIV. The Board of Dental Examiners created by this Act may sue or be sued, and it shall be made a party under the name of the Board of Dental Examiners of the State of Michigan in all actions brought by or against it.

SEC. XV. This Act shall take effect and be in force from and after its passage.

MANNING A. BIRGE, Chairman.
J. WARD HOUSE,
BEN. H. LEE,
*Committee of Grand Rapids Dental Society.*

